# Immunologic hierarchy and promiscuity of melanoma helper peptides

**DOI:** 10.1186/2051-1426-1-S1-P104

**Published:** 2013-11-07

**Authors:** Yinin Hu, Gina R Petroni, Walter C Olson, Andrea Czarkowski, Mark Smolkin, William W Grosh, Patrice K Rehm, Cheryl F Murphy, Elizabeth J Coleman

**Affiliations:** 1 University of Virginia, Charlottesville, VA, USA

## Background

Melanoma vaccines have been designed to expand specific CD8+ T-cells, but melanoma-reactive helper T-cells also can have antitumor activity. We previously observed clinical activity of a vaccine incorporating 6 melanoma helper peptides (6MHP), and found associations between CD4+ T cell response and survival. With the present study, in the spirit of personalized cancer immunotherapy, we define the relative immunogenicity and HLA allele promiscuity of individual helper peptides, and identify helper peptide-mediated augmentation of melanoma-specific CD8+ T-cell response.

## Methods

Thirty-seven patients with stage IIIB-IV melanoma were vaccinated with 6MHP in incomplete Freund’s adjuvant. The vaccines contained 6 peptides: gp10044-59 (first 3 amino acids: WNR), Tyrosinase56-70 (AQN), Tyrosinase386-406 (FLL), Melan-A/MART-151-73 (RNG), MAGE-A3281-295 (TSY), and MAGE-A1, 2,3,6121-134 (LLK). Peripheral blood mononuclear cells (PBMC) and sentinel immunized nodes (SIN) were collected. CD4+ T-cell proliferation was assessed by thymidine uptake after exposure to peptides. CD8+ T-cell response was assessed by direct IFN-γ ELIspot assay against 14 MHC class I-restricted peptides.

## Results

**Figure 1:**
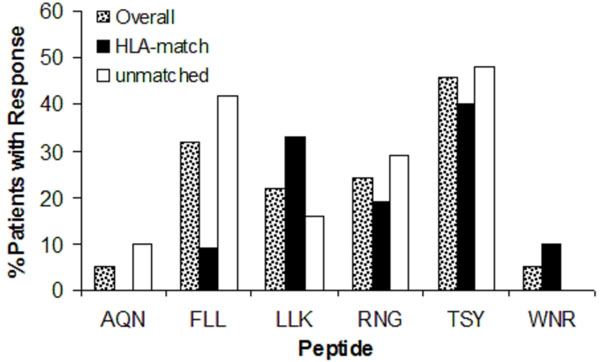


Vaccines induced CD4+ T cell responses to the 6MHP pool in 78% (29/37) of patients in SIN and 57% (21/37) in PBMC for an overall response rate of 81% (30/37), with responses to an average of 2 peptides per patient. The two most frequently immunogenic peptides were TSY at 49% (18/37) and FLL at 32% (12/37). HLA restriction was not limited to alleles originally described; for each peptide, the proportion of immune responsive patients whose HLA-DR expression matched those originally described was similar to the proportion of patients with different HLA-DR expression (unmatched, Figure 1). Vaccine-associated CD8+ T-cell response was observed in 45% (5/11) of patients, and three of these patients demonstrated CD4+ T-cell response toward TSY.

## Conclusions

The 6MHP vaccine has CD4+ T-cell immunogenicity beyond known HLA-DR restrictions. Patients whose tumors express tyrosinase, MAGE-A3, and several other MAGE proteins may be ideal for vaccination with 6MHP. The 4 most immunogenic peptides warrant further study, perhaps in combination immune therapies.

